# A novel cuproptosis-related lncRNA signature predicts prognosis and therapeutic response in bladder cancer

**DOI:** 10.3389/fgene.2022.1082691

**Published:** 2023-01-04

**Authors:** Jinming Cai, Haoran Xie, Yilin Yan, Zhengnan Huang, Pengfei Tang, Xiangqian Cao, Zeyi Wang, Chenkai Yang, Jiling Wen, Mingyue Tan, Fang Zhang, Bing Shen

**Affiliations:** ^1^ Department of Urology, Shanghai General Hospital, Shanghai Jiao Tong University School of Medicine, Shanghai, China; ^2^ Department of Urology, Shanghai East Hospital, Tongji University School of Medicine, Shanghai, China; ^3^ Department of Gastroenterology, Shanghai General Hospital, Shanghai Jiao Tong University School of Medicine, Shanghai, China; ^4^ Department of Urology, Shanghai General Hospital Affiliated to Nanjing Medical University, Shanghai, China; ^5^ Department of Urology, Shuguang Hospital, Shanghai University of Traditional Chinese Medicine, Shanghai, China

**Keywords:** cuproptosis, bladder cancer, lncRNAs, immunotherapy, survival analysis

## Abstract

Bladder cancer (BC) ranks the tenth in the incidence of global tumor epidemiology. LncRNAs and cuproptosis were discovered to regulate the cell death. Herein, we downloaded transcriptome profiling, mutational data, and clinical data on patients from The Cancer Genome Atlas (TCGA). High- and low-risk BC patients were categorized. Three CRLs (AL590428.1, AL138756.1 and GUSBP11) were taken into prognostic signature through least absolute shrinkage and selection operator (LASSO) Cox regression. Worse OS and PFS were shown in high-risk group (*p* < 0.05). ROC, independent prognostic analyses, nomogram and C-index were predicted *via* CRLs. Gene Ontology (GO) and Kyoto Encyclopedia of Genes and Genomes (KEGG) analysis indicated IncRNAs play a biological role in BC progression. Immune-related functions showed the high-risk group received more benefit from immunotherapy and had stronger immune responses, and the overall survival was better (*p* < 0.05). Finally, a more effective outcome (*p* < 0.05) was found from clinical immunotherapy *via* the TIDE algorithm and many potential anti-tumor drugs were identified. In our study, the cuproptosis-related signature provided a novel tool to predict the prognosis in BC patients accurately and provided a novel strategy for clinical immunotherapy and clinical applications.

## Introduction

One of the most common malignancies of the urogenital tract is bladder cancer (BC). According to the latest epidemic study of tumor incidence, BC ranks tenth (Sung et al., 2021). The clinical pathology of BC contains urothelial carcinoma (90–95%), adenocarcinoma and squamous cell carcinoma (Lenis et al., 2020). Based on pathological classification, tumor invades the muscle or beyond, and invades the urothelium or lamina propria, which were called muscle-invasive bladder cancer (MIBC), and non–muscle-invasive bladder cancer (NMIBC), respectively (Hurst et al., 2018). The rate of 5-year recurrence is 50–70%, with a risk of progression of 10–30%, after receiving the initial transurethral resection of bladder tumor (TURBT) (Martinez Rodriguez et al., 2017). Therefore, owing to the high incidence, mortality and recurrence of BC, it is necessary to construct more prognostic models. Long non-coding RNSs (lncRNAs) can regulate the expression of oncogene or cancer related genes, which are 200 nucleotides of non-coding transcripts in length. Previous studies have indicated lncRNAs were enriched in many biological processions, such as immune responses, metabolism regulation, and cell metastasis (Zhu et al., 2021). LncRNAs have been found to have specific relevance to the pathogenesis of BC and influence the progression of BC (Li et al., 2020).

Copper plays a crucial role in maintaining biological processes in various life entities. Recent researches found the copper concentration of cancer patient is significantly higher than the health in serum and cancer tissue (Ishida et al., 2013; Blockhuys et al., 2017; Ge et al., 2022). Given the copper disorder may lead to metabolic imbalance and cytotoxicity, copper variation of intracellular levels may influence tumor progression and development (Babak and Ahn, 2021). Moreover, copper chemical materials (copper chelators, ionophores, etc) have been utilized to anticancer treatment (Brady et al., 2017; O'Day et al., 2013). A recent research uncovered a new cell death pathway, cuproptosis, in copper compounds coupled with lipoylated tricarboxylic acid (TCA) cycle components, resulting in cell death and toxic protein stress, directly (Tsvetkov et al., 2022). BC showed enhanced glucose utilization for glycolysis, and pathways significantly unbalanced in tumor relative to normal urothelium included tricarboxylic acid (TCA) cycle, glucose and so on (Sahu et al., 2017). A vital role for glutamine (Gln) from TCA in promoting proliferation of Gln-dependent BC T24 cell line has been documented, which supplemented adenosine triphosphate (ATP) generation and neutralizing reactive oxygen species (ROS) for activating the STAT3 pathway (Sun et al., 2019a). Several genes were identified connected with copper-induced cell death, which can offer a chance new to construct a new prognosis model.

**FIGURE 1:**
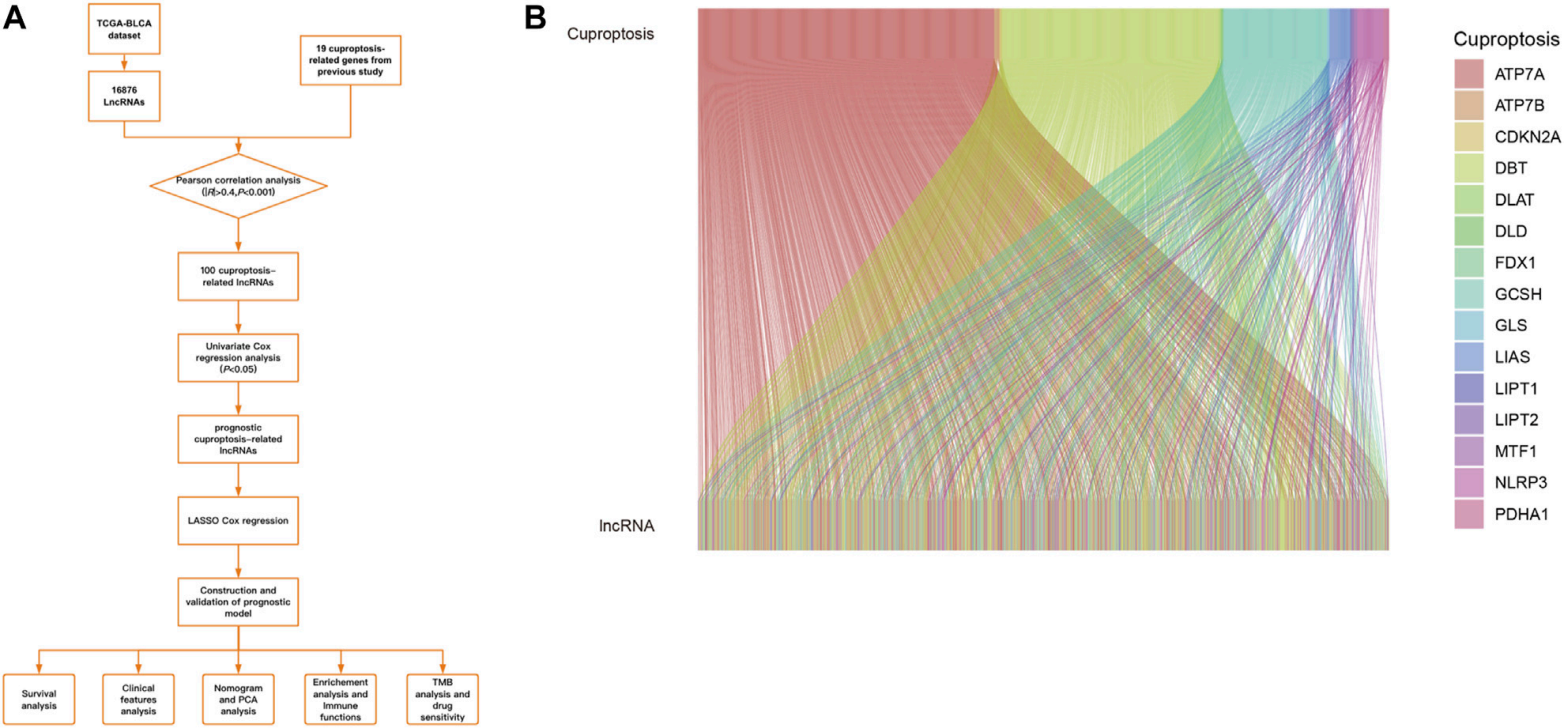
**(A)**.Workflow of our study. **(B)**.The results of CRGs and CRLs co-expression in Sankey diagram.

In our study, an emerging prognostic model for BC was explored based on CRLs that can offer prognosis prediction and selection of patients for immunotherapies. The workflow was presented in Figure 1A. The prognosis model was validated *via* bioinformatics. Moreover, functional enrichment analysis was performed to discuss potential pathways for signaling in groups at risk. In the end, immune functions and immunotherapy were analyzed in the risk group.

## Materials and methods

### Data collection and preprocessing

The expression of genes and clinical data were downloaded from TCGA database up to 29 March 2022 (https://portal.gdc.cancer.gov/repository). Then, Active Perl was used to pre-process the raw data of gene expression and human configuration data and synthetic matrices were obtained. LncRNAs data and mRNAs data were distinguished *via* Active Perl. Moreover, clinical data was prepared to estimate missing overall survival (OS) values. In the end, a total of 431 patients’ gene expression profiles were obtained. And using the “caret” R package, train risk is divided by test risk in a 1:1 ratio. The focal-level copy number variation (CNV) values were downloaded *via* GISTIC2, using a “masked copy number fragment” file. (Mermel et al., 2011).

### Identification of cuproptosis-related genes and lncRNAs in TCGA cohort

A previous study obtained cuproptosis-related genes (CRGs) (Supplementary Table S1) (Tsvetkov et al., 2022). Using “R” (version 4.1.0) software, CRLs were distinguished *via* “limma” R package (|Pearson R|>0.4, *p* < 0.001) (Supplementary Table S2). CRGs were isolated from the whole gene expression data (Supplementary Table S3).

### Construction of CRLs prognostic signature

956 prognostic CRLs were identified *via* univariate Cox regression analysis (*p* < 0.05) with “Survival” R package (Eaton et al., 2020). The gene signature was identified, which contains most like biomarkers of prognosis *via* “glmnet” R package (Friedman et al., 2010). LASSO with a tenfold cross-validation offered a tool to establish the prognostic model. Based on the following formula:

$$Risk\;score=\sum \left(Coef*\mathrm{EXP}\right),$$

The risk score was calculated. *Coef* means the coefficient and *EXP* means the expression level of each prognostic CRLs in the formula. Based on risk scores, High- and low-risk BC patients were categorized. To elucidate the predictive value of the CRLs-based model of prognostic signature, we performed the receiver operating characteristic (ROC) of 1-year survival in the training and testing group by “ROC” R package (Blanche et al., 2013). In accordance with the optimal risk cutoff value analyzed by “Survival” R package, the patients were categorized as high-risk or low-risk (Eaton et al., 2020). The prognostic value of two-CRL signature on BC was analyzed by *Kaplan-Meier*. Then, the risk model was tested using analyses of Cox regressions, univariate and multivariate, to estimate if the risk model showed better predictive ability of prognosis independently associated with other clinicopathological features, such as gender, grade and pathologic staging. Moreover, a TCGA-based prognostic nomogram was developed. Clinical parameters and independent prognostic factors are included in the nomogram.

### Construction of risk score model

Basing on median values, we divided samples of all patients into high- and low-subgroups to estimate the prognosis and the signature. Progression-free survival (PFS) and OS were calculated by “Survival” R package. We estimated independent prognostic abilities of risk prediction models using both univariate and multivariate analyses. CRLs expression and patient survival status were shown based on the risk scores *via* “Pheatmap” R package and pheatmap. C-Index was showed with “Survival” R package for validating the performance of the model to predict patients’ survival.

### Nomogram construction and clinical feature validations

To exhibit the difference between the actual and predicted results, the nomogram was constructed for gender, grade, stage and the calibration curves were plotted. 1-, 3- and 5-year OS were predicted *via* a stepwise Cox regression in the TCGA dataset.

### Analysis of principle component analysis (PCA) and enrichment function

The “limma” R package and “Scatterplot3d” R package were utilized to construct PCA, which can show clear distribution in different risk groups. The KEGG and GO enrichment of CRLs was performed with the “ClusterProlfiler” R package.

### TMB and immune-related functional analysis

The correlation between TMB and risk score was completed by “maftools” R package. The difference between patient survival status and TMB was explored *via* the “Survival” R package. Immune-related functions were analyzed and its differences were identified *via* “limma” and “GSVA” R package, which were visualized by “Pheatmap” R package.

### Analyses of immunotherapy and potential pharmaceuticals

The Tumor Immune Dysfunction and Exclusion (TIDE) database (http://tide.dfci.harvard.edu/) contained tumor pre-treatment expression profiles. BC’s TIDE dataset was downloaded and the correlation between TMB and risk score was explored by “ggpubr” and “limma” R packages. The “pRRophetic”, “ggpubr” and “ggplot2” R package were conducted to screen for potential therapeutic drugs and explore the sensitivity of drugs.

### Statistical analysis

R software and packages were used for statistical analysis. Here, based on False Discovery Rates (FDRs) < 0.05 and the *p*-value (<0.05), statistics were collected. *Kaplan-Meier* (KM) was utilized to compare OS between subgroups. *Pearson* correlation tests were used to identify CRLs. The *Chi-square* test was conducted to analyze categorical variables of groups. The differential risk scores of subgroups were compared *via* the *Student’s t*-test. Moreover, the prognostic ability of risk scores and other clinical data was explored using multivariate and univariate Cox regression analyses. Herein, statistical significance was determined at *p < 0.05; **p < 0.01; ***p < 0.001.

## Results

### Identification of bladder cancer-specific CRLs and establishment of the prognostic signature

**FIGURE 2:**
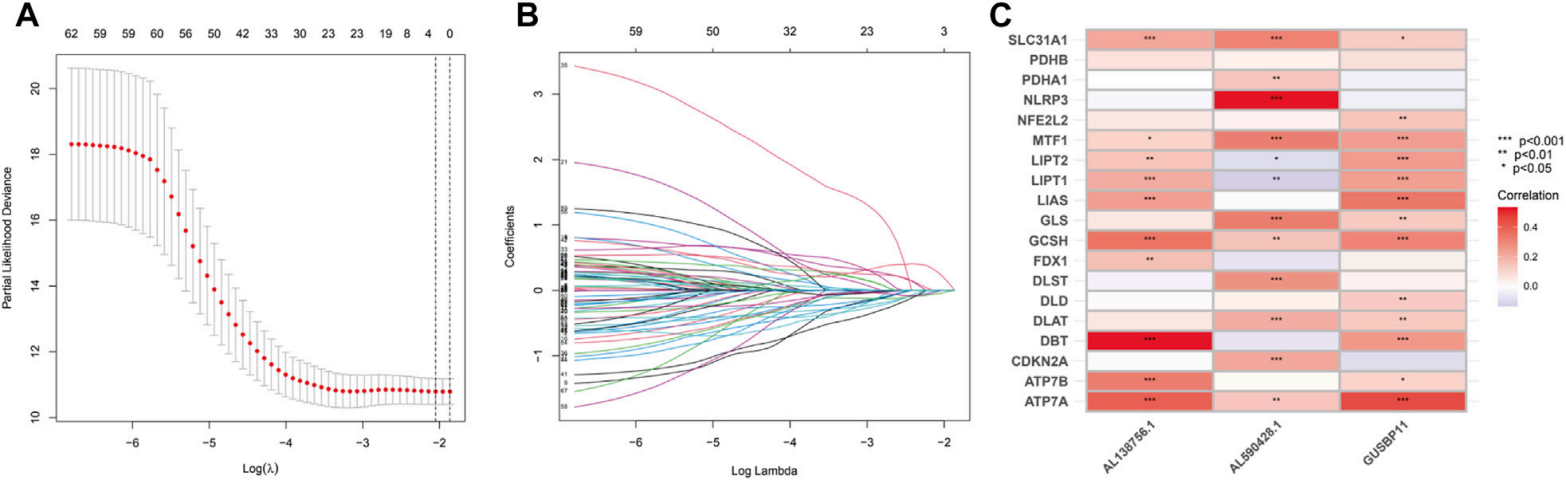
Identification of the CRLs. **(A)**.LASSO regression of prognotic CRLs. **(B)**.The penalty parameter (ƛ) in the LASSO regression model was selected through two cross-validation. **(C)**.The relationship between CRLs and CRGs for the signature in the corrlation heatmap.

TCGA-BLCA dataset was downloaded, which contains 412 cancer samples and 19 normal samples. Clinical features were exhibited in the Table 1. 956 CRLs were identified from 16876 lncRNAs and 19 CRGs that met the criteria (|R|>0.4 and *p* < 0.001). Sankey diagram was plotted to analyze the co-expression relationship between CRGs and CRLs (Figure 1B). LASSO Cox regression was utilized to identify CRLs in the training group (Figures 2A, B). A total of 69 CRLs were identified through univariate Cox regression (Supplementary Figure S1). Low- and high-risk lncRNAs were shown with red and green in the forest plot, respectively. Then, 3 CRLs as independent prognostic factors were identified *via* multivariate Cox regression (*p* < 0.05). According to the formula and the expression levels of the 3 lncRNAs, the risk score of all samples was obtained. Risk score = (-0.278725960360529* AL138756.1) + (+1.03981953291805* AL590428.1)+(-0.454158106791872* GUSBP11). Finally, the correlation heatmap was used to show the relationship of between CRGs and lncRNAs (Figure 2C).

**TABLE 1:** Clinical features of BC patients in TCGA dataset.

Age		
	>65	162
	≦65	250
Gender		
	Male	304
	Female	108
Grade		
	High grade	388
	Low grade	24
Stage		
	Stage Ⅰ-Ⅱ	133
	Stage Ⅲ-Ⅳ	277
	unknow	2
T stage		
	T0	1
	T1	3
	T2	120
	T3	196
	T4	59
	unknow	33
N stage		
	N0	239
	N1	47
	N2	76
	N3	8
	unknow	42
M stage		
	M1	11
	M0	196
	unknow	205

### The correlation of survival status and genetic signature

**FIGURE 3:**
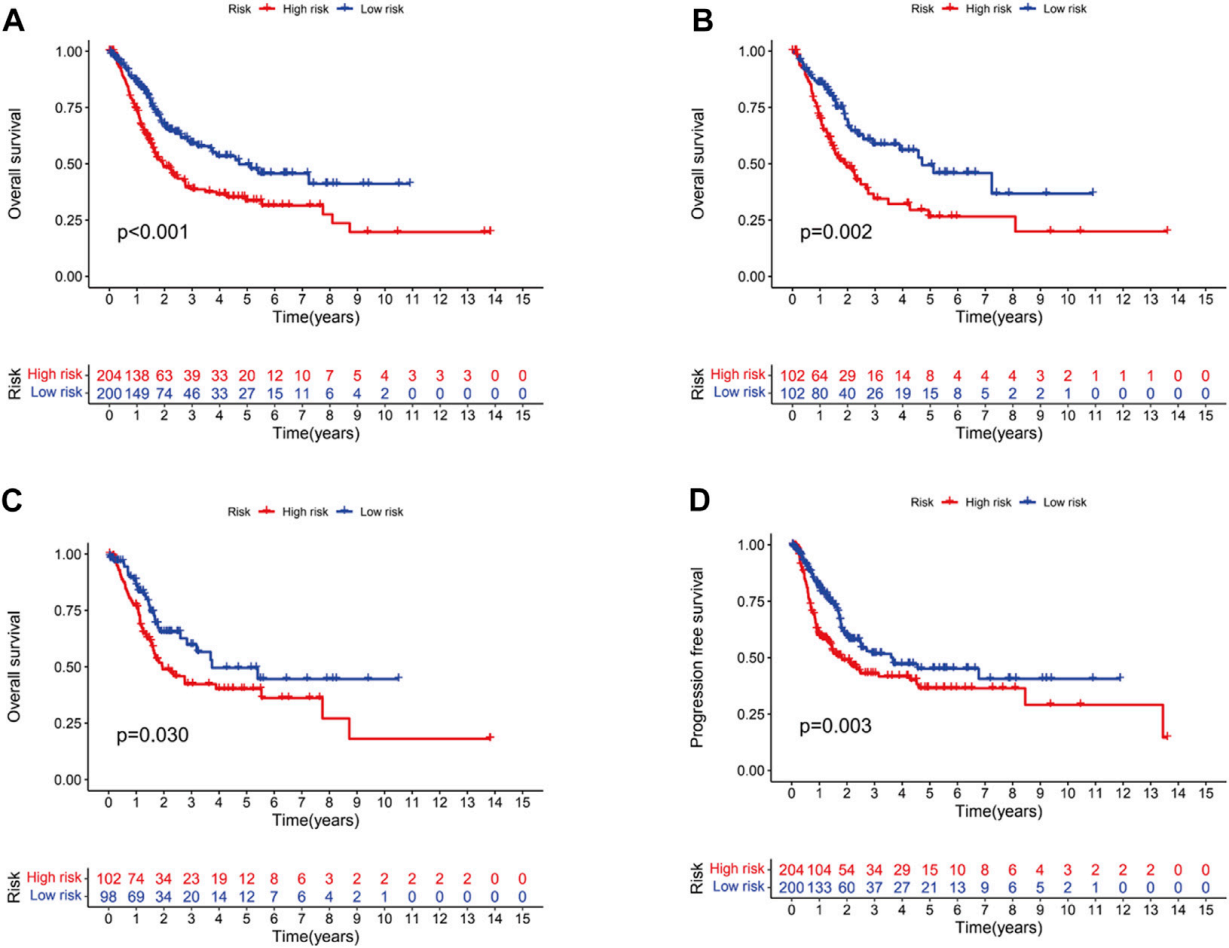
Kaplan-Meier survival analysis of BC patients. Overall survival of BC patients in all groups **(A)**, train groups **(B)**, testing groups **(C)**, respectively. **(D)**.PFS of BC patients in all groups.

**FIGURE 4:**
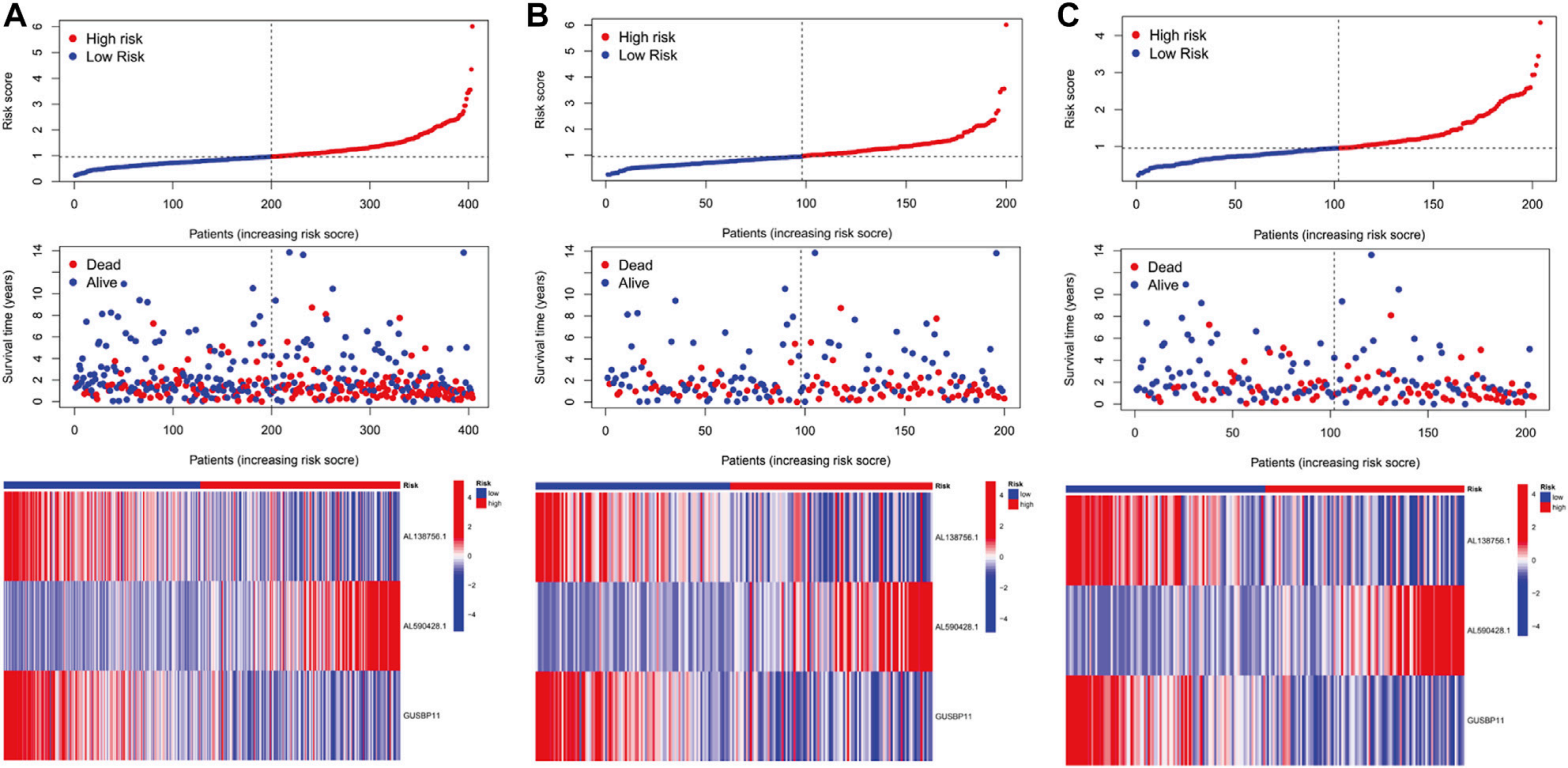
The predictive performance of characteristics. Distribution of risk score, status and the expression of prognostic CRLs in the all group **(A)**, training group **(B)**, testing group **(C)**.

In the light of the median risk scores as the cut-off value, low- and high-risk patients were classified. OS and PFS in high-risk group were found to be significantly shorter than that in low-risk group in three groups (all groups, training group and testing group) (*p* < 0.05) (Figures 3A–D). The risk curves were visualized to exhibit the correlation between risk score and survival status in subgroups (all groups, training group and testing group), which were displayed in Figures 4A–C. Moreover, mortality was higher in high-risk groups than in low-risk groups (*p* < 0.05). The heatmap was utilized to show the differences of two risk subgroups for 3 lncRNAs with good consistency. AL138756.1 and GUSBP11 were overexpressed in the low- subgroup and underexpressed in the high- subgroup, while AL590428.1 showed the opposite trend. Therefore, it was found that AL138756.1 and GUSBP11 expression are negatively correlated with risk scores. AL590428.1 showed the positive correlation with risk scores.

### Univariate and multivariatae analysis of the prognostic model

**FIGURE 5:**
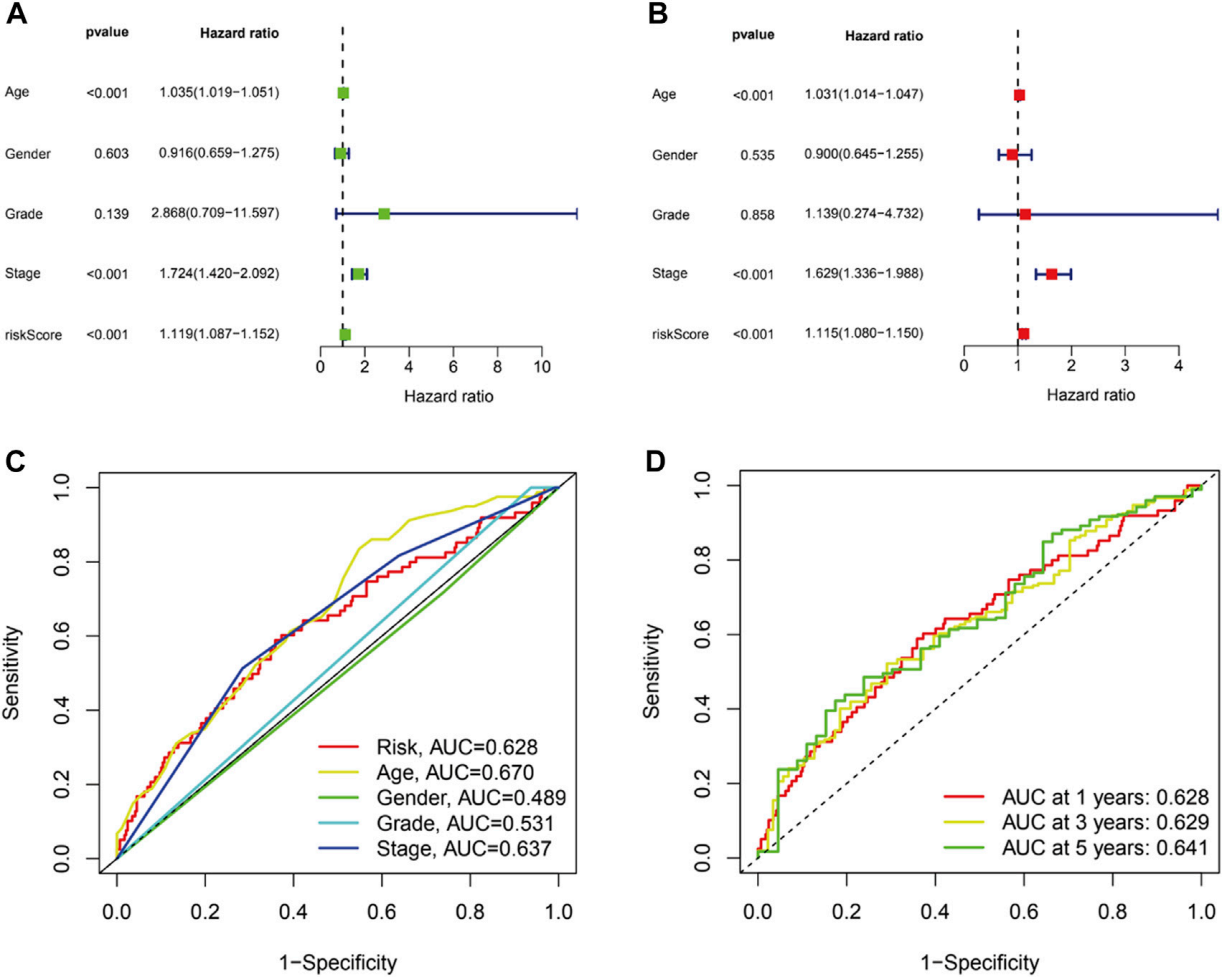
The prognostic value of the CRLs in BC patients. Univariate **(A)** and multivariate **(B)** independent prognostic analysis to explore the risk score was independently related with OS. **(C)**.ROC curve analysis was utilized to compare the predictive accuracy between the risk model and various clinical features. **(D)**.ROC curves for 1-, 3-, and 5-year survival prediction.

To figure out whether the signature we constructed could be utilized to be independent prognostic factors, univariate and multivariate Cox regression were conducted. The results indicated that age (HR = 1.035, 1.019–1.051, *p* < .001), stage (HR = 1.724, 1.420–2.092, *p* < .001) and risk score (HR = 1.119, 1.087–1.152, *p* < .001) were significantly associated with OS in univariate Cox regression (Figure 5A). And multivariate Cox regression suggested age (HR = 1.031, 1.014–1.047, *p* < .001), stage (HR = 1.629, 1.336–1.988, *p* < .001) and risk score (HR = 1.115, 1.080–1.150, *p* < .001) were independently related with OS (Figure 5B), which suggested the prognostic model can be regarded as an independent prognostic indicator for BC patients. Then, to estimate the predictive power of the risk score, ROCs were performed. The area under curve (AUC) of the risk score was 0.628 (Figure 5C). Similarly, AUCs for 1, 3, and 5 years were 0.628, 0.630, and 0.641 (Figure 5D). AUCs were greater than or equal to 0.600, which suggested the prognostic model has a better diagnostic value.

### Construction and validation of the OS nomogram and principal component analysis

**FIGURE 6:**
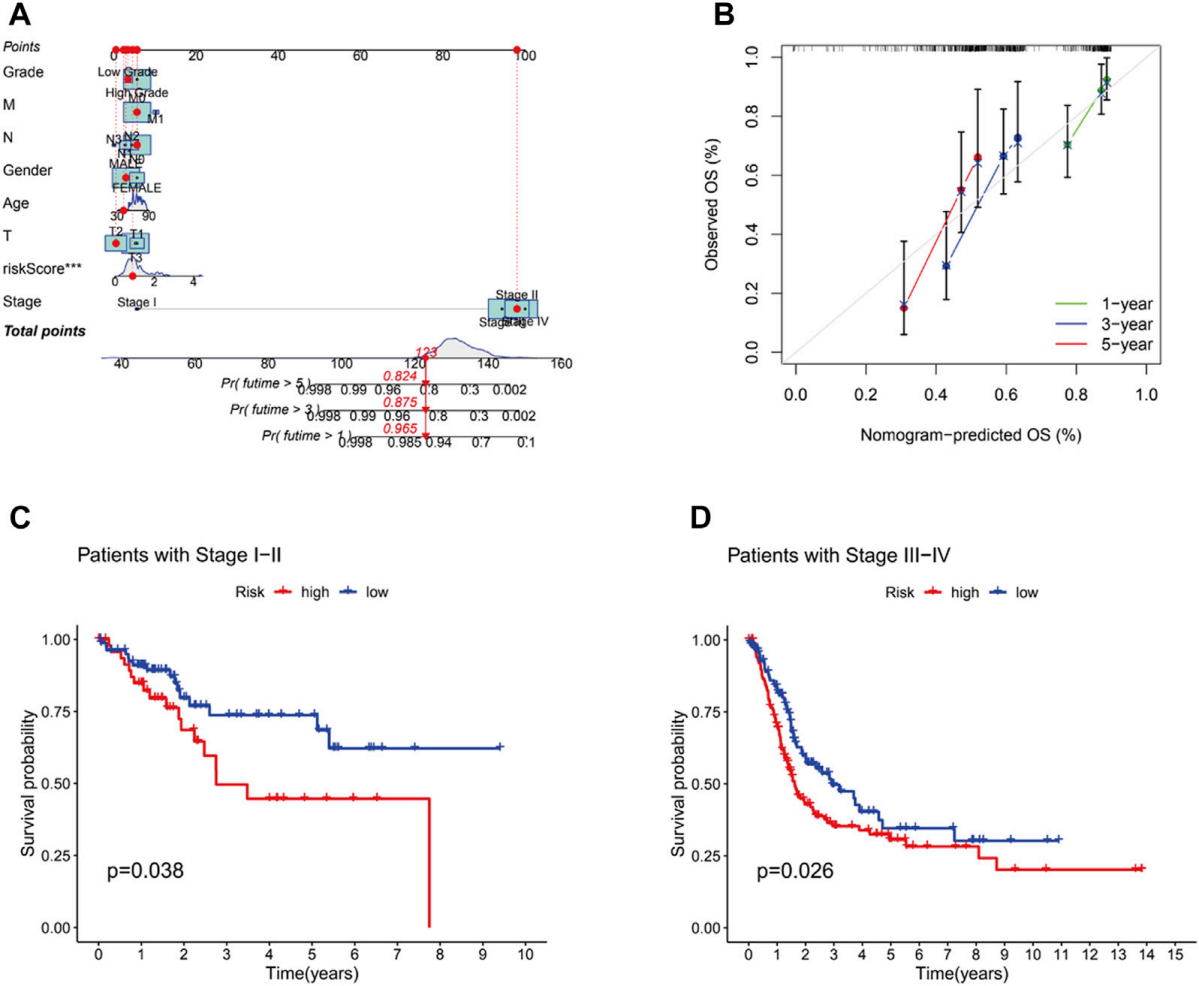
The construction of nomogram and the predictive outcomes of clinical subgroups. **(A)**.The combined nomogram for the risk model and other clinicopathological factors. **(B)**.Calibration curves of 1-, 3-, and 5-year. The high and low risk groups were extimated to analyze the survival influence at stage Ⅰ-Ⅱ **(C)** and stage Ⅲ-Ⅳ **(D)**.

**FIGURE 7:**
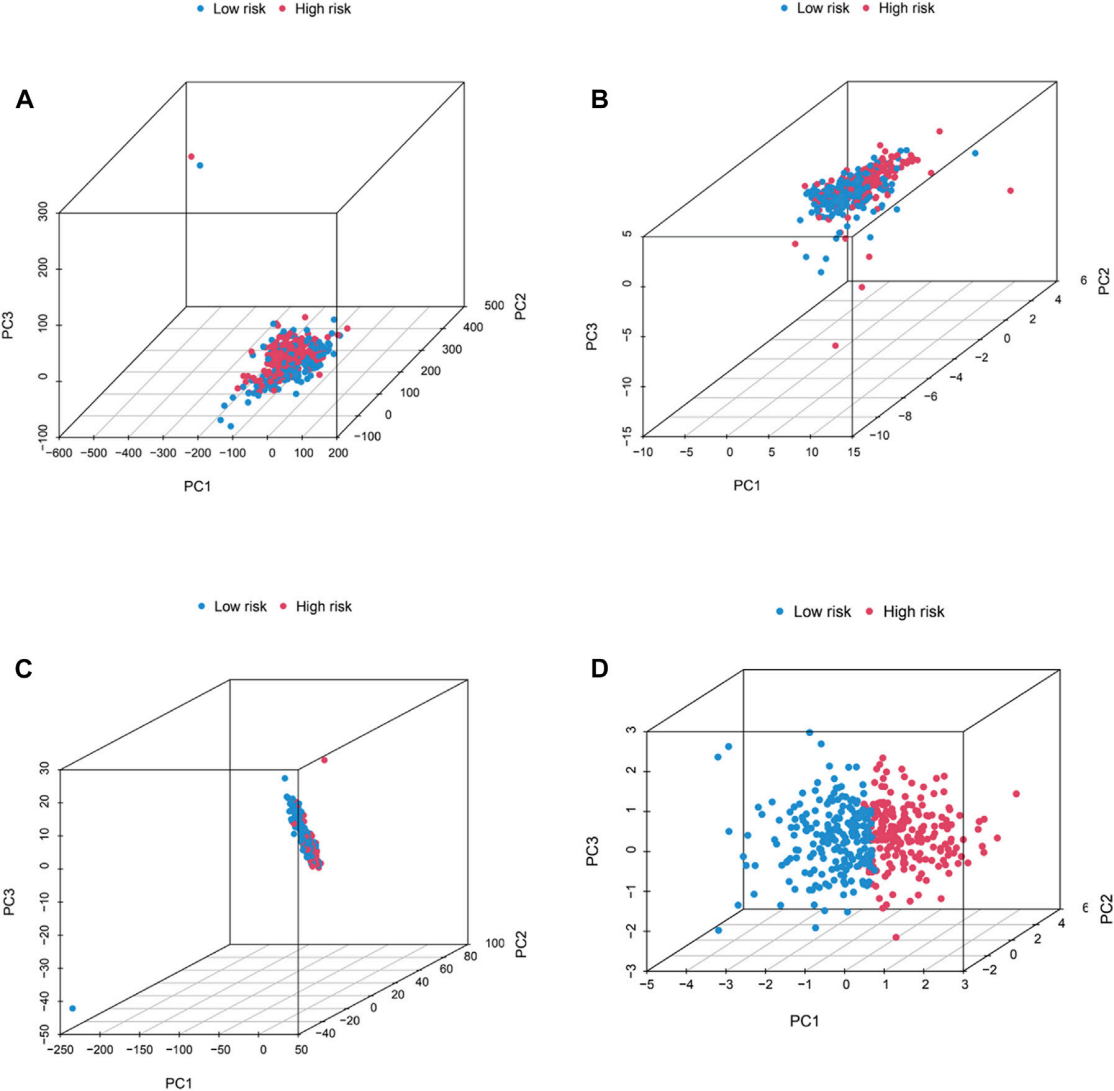
PCA analysis. The distribution of BC patients in all genes **(A)**, CRGs **(B)**, CRLs **(C)**, and risk lncRNAs **(D)** *via* PCA analysis.

A nomogram, including grade, gender, age, T stage, N stage, M stage, stage and risk, was plotted and the nomogram predicted the survival of 1-, 3- and 5-year (Figures 6A, B). Then, C-index curves were plotted to determine if there were any discrepancies in patients’ survival over time (Supplementary Table S2). The results indicated that the risk signature has a high predictive accuracy of survival status in BC patients and was not influenced by different clinical grades. Moreover, OS of clinical stage in subgroups was analyzed. As shown in Figures 6C, D, OS in stages I-IV differed significantly in the low- and high-risk individuals (*p <* .05). At last, the distribution of all genes, CRGs, CRLs and risk lncRNAs was analyzed *via* PCA (Figure 7), which showed a clear status of risk lncRNAs. Therefore, the results demonstrated all lncRNAs were reliably utilized to establish the signature.

### Functional enrichment and immune-related functional analyses

**FIGURE 8:**
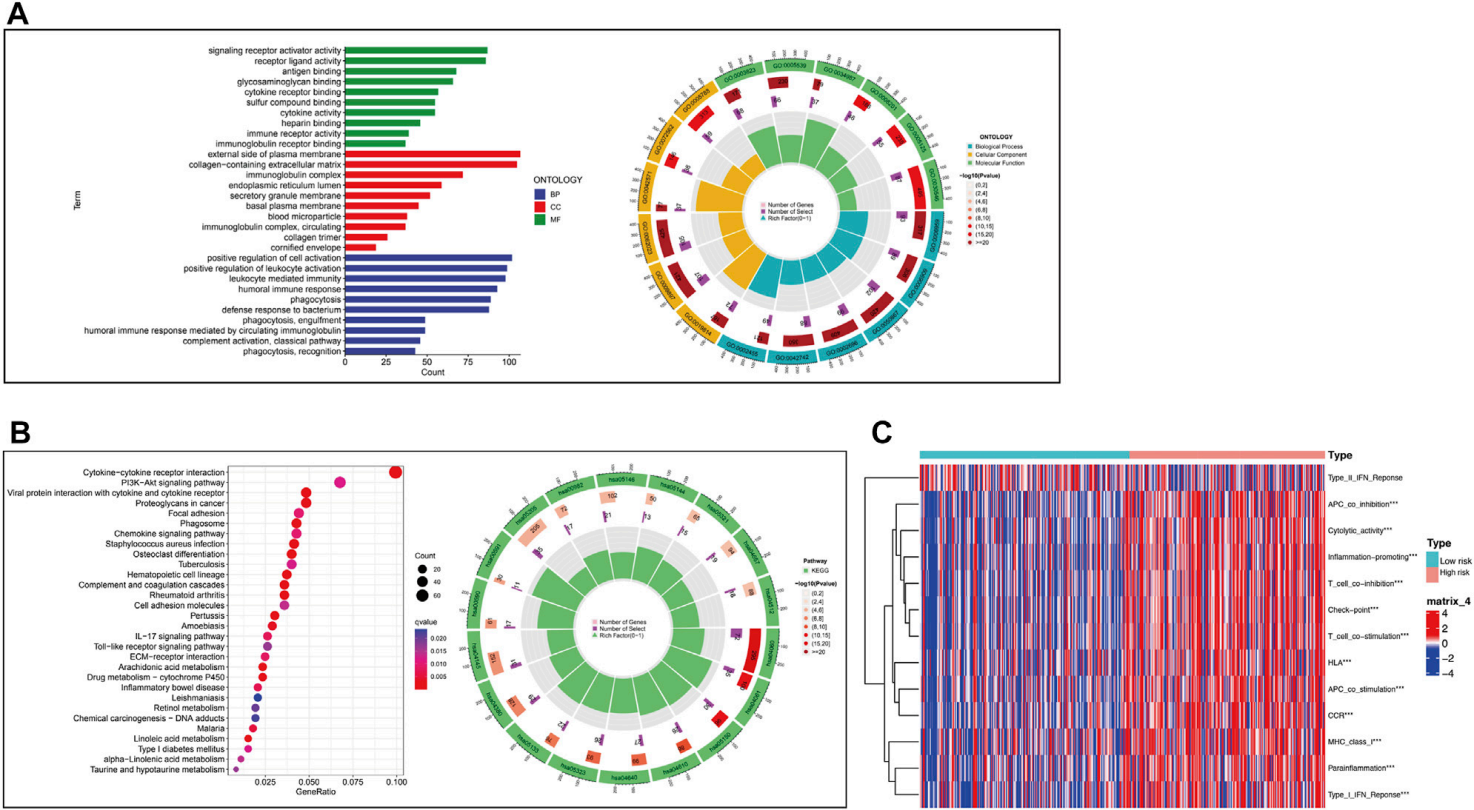
Functional enrichment analysis and immune-related functional analysis. **(A)**.GO enrichment of CRLs and numeric details. **(B)**.KEGG enrichment of CRLs and numeric details. **(C)**.Immune-related functions of CRLs.

To understand the relationship between the CRLs and tumor development, GO and KEGG were performed (Figures 8A, B). The results of GO results showed CRLs were enriched in signaling receptor activator activity, external side of plasma membrane, and positive regulation of cell activation, which were molecular function, cellular component, and biological procession (*p* < .05), respectively. KEGG results indicated these lncRNAs may be associated with cytokine-cytokine receptor interaction, viral protein interaction with cytokine and cytokine receptor, PI3K-Akt signaling pathway and so on (*p* < .05). Circle plot showed the distribution of CRLs in GO and KEGG enrichment. Furthermore, immune-related functions were analyzed to figure out the immune status of subgroups (Figure 8C). The results showed there was a significant increase in Type_I_IFN_Response, Parainflammation, APC co-stimulation, T cell co-stimulation, T cell co-inhibition, inflammation-promoting, cytolytic-activity and so on in the high-risk group when compared with the low-risk group (*p* < .05).

### The burden of tumor mutations and drug sensitivity of tumor

**FIGURE 9:**
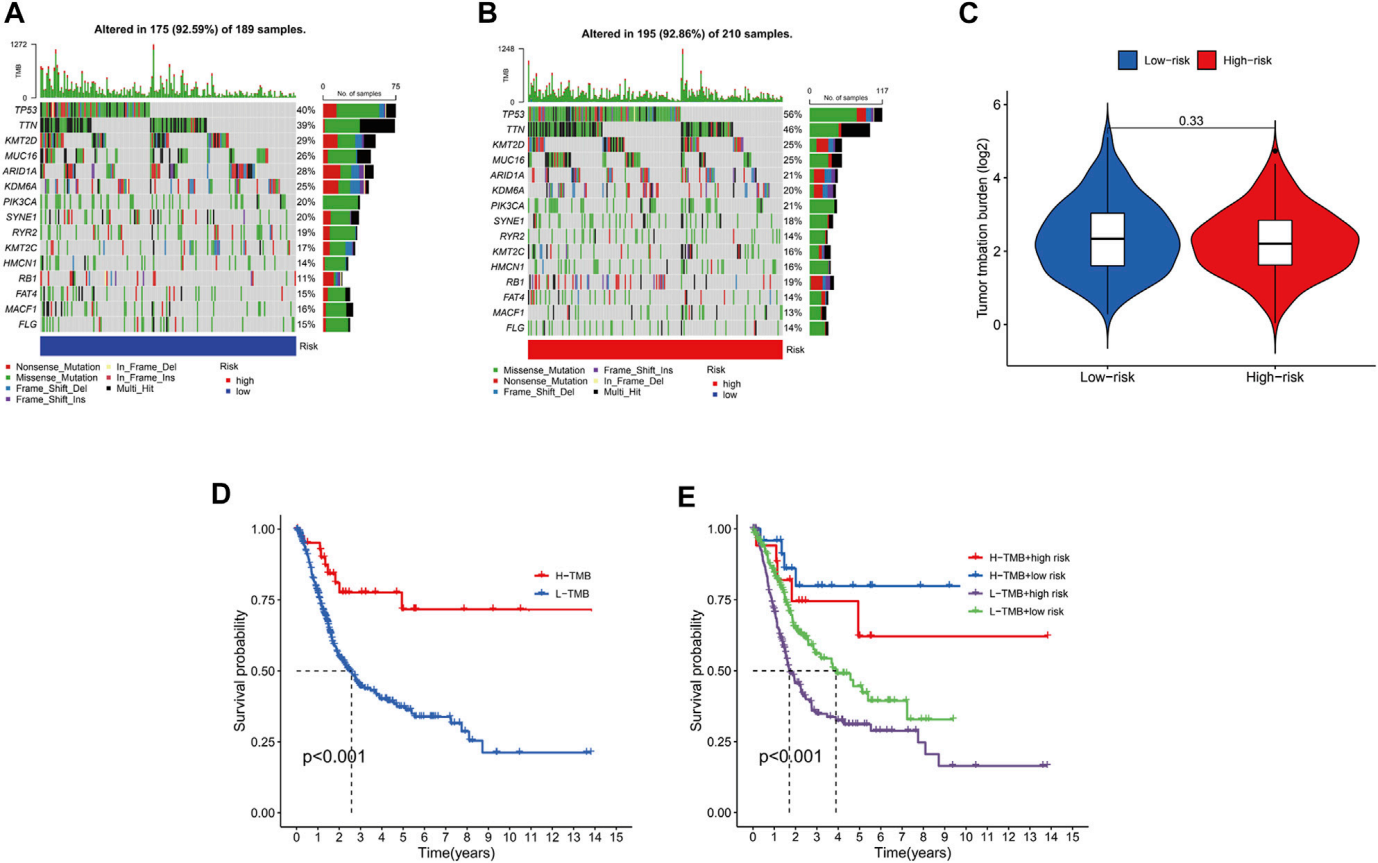
The relationship between the risk signature and TMB. Top 15 mutation genes of BC for the low-risk **(A)**, high-risk **(B)** groups in waterfall plot. **(C)**.Different TMB levels in subgroups in BC. **(D)**.Survival status of high TMB levels and low TMB levels in BC. **(E)**.Survival curves for combined TMB-risk subgroups in BC.

**FIGURE 10:**
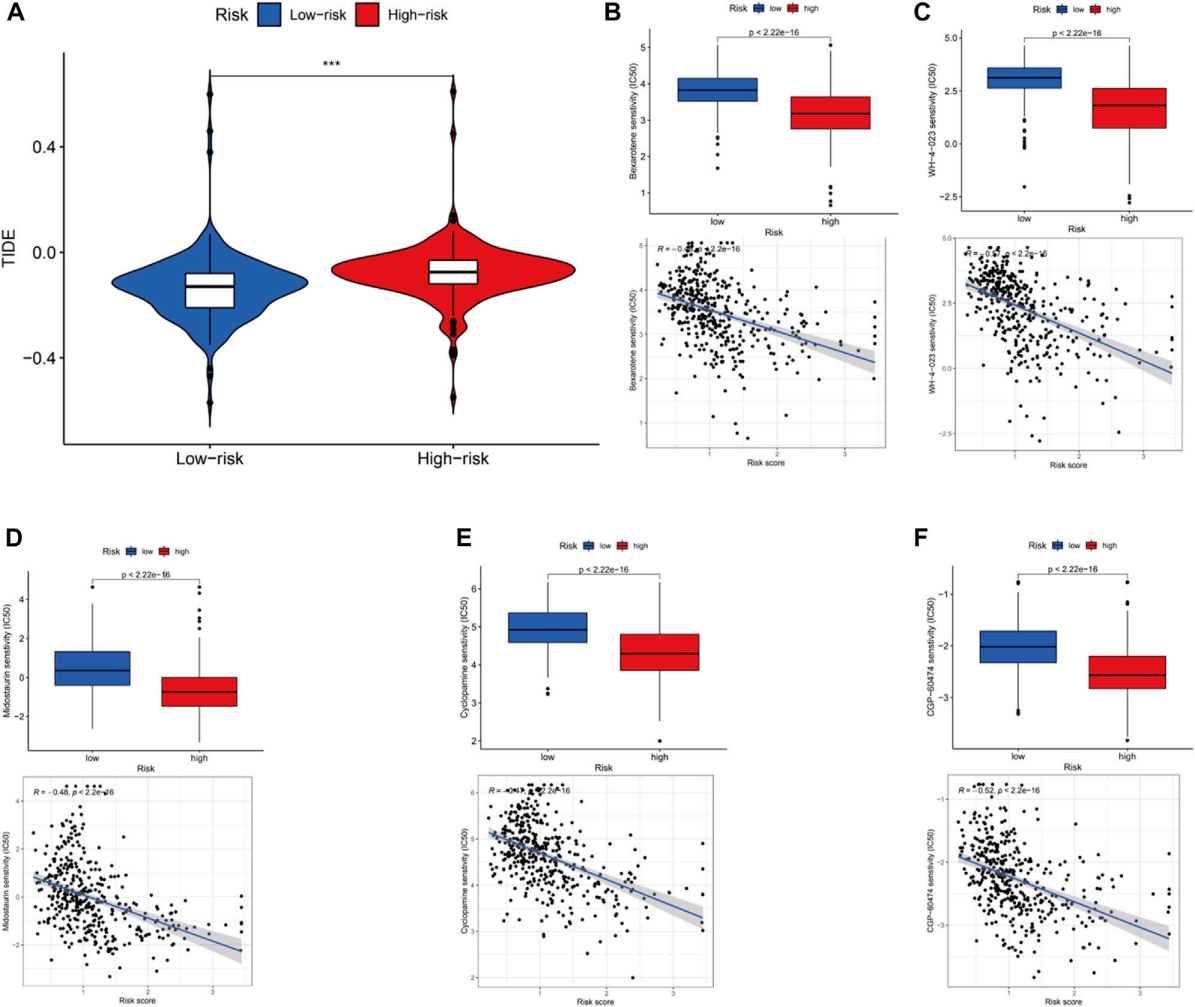
Immunotherapy, drug sensitivity and the relationship between the drug sensitivityand risk scores. **(A)**.Different TIDE in the high and low groups. Observed the drug sensitivity and its relationship with risk scores in top 5 drugs: Bexarotene **(B)**, WH-4-023 **(C)**, Midostaurin **(D)**, Cyclopamine **(E)** and CGP-60474 **(F)**.

Mutation of most genes was observed in the low- and high-risk groups *via* maftools algorithm. The mutation of TP53 (high risk, 56%; low risk 40%) and TTN (high risk, 46%; low risk, 39%) in the high-risk group was more common than that in the low-risk one in the top-5 genes with highest mutation (*p* < .05). The mutation of KMT2D (high risk, 25%; low risk 29%), MUC16 (high risk, 25%; low risk, 26%) and ARID1A (high risk, 21%; low risk, 28%) in the low-risk group was higher than that in the high-risk group in the top-5 genes with highest mutation (*p* < .05) (Figures 9A, B). Moreover, it was no significant to distinguish low-risk from high-risk groups (*p* = 0.33) (Figure 9C). Then, to determine whether there was any association between the TMB and the BC survival status, survival curves were plotted in pure TMB and combined TMB-risk groups (Figures 9D, E). There was a significant difference among subgroups (*p* < 0.05). High TMB had a longer survival time than low TMB (*p* < 0.05). Meanwhile, comparisons of all subgroups were significantly different (*p* < 0.05). Furthermore, the TIDE algorithm was utilized to investigate the difference of sensitivity to immunotherapy in two subgroups (Figure 10A). The results indicated the TIDE score in the high-risk group was higher than that in the low-risk group (*p* < 0.05). Finally, potential anti-tumor drugs were screened *via* the algorithm from the “pRRophetic” R package. The top 5 most significantly associated drugs were listed and an analysis of the correlation between sensitivity and risk scores was conducted (*p* < 0.05), including Bexarotene, WH-4-023, Midostaurin, Cyclopamine and CGP-60474 (Figures 10B–F).

## Discussion

BC is notorious cancer with high incidence and mortality. Previous studies have found the Nuclear Matrix Protein 22 (NMP22) and cytokeratin markers (CYFRA 21-1 or UBC), which can play a role in biological markers of BC as a substitute to urinary cytology (Sánchez-Carbayo et al., 2000). However, no available molecular biomarker can replace cytology in sensitivity and specificity. Therefore, a reliable BC risk model was constructed, which is an aggressive need to figure out the clinical outcomes of BC patients. For the extension of multi-omics data and database, optimized data mining algorithms have an important influence on tumor research (Angus et al., 2019; Nacev et al., 2019; Liu et al., 2020; Tabassum et al., 2020). Multiple risk signatures and transcriptome profiling provided a novel insight into the prognosis of individual patients *via* combining the gene expression and clinical features (Xie et al., 2021; Yan et al., 2021). Due to the lack of systematic evaluation, most studies cannot be utilized to clinical practice. Hence, it is urgent that prognostic factors of BC patients are identified to distinguish the high-risk population.

A series of researches have reported the relationship between copper and BC (Konukoğlu et al., 1996; Lin et al., 2009; Mazdak et al., 2010; Gecit et al., 2011; Guo et al., 2012). Moreover, copper complex [Cu ^II^_2_Cu ^I^(L)_2_(Br)_3_] have been found to succeed inducing apoptosis in pancreatic cancer, such as tolfenamic acid–Cu II) complex and Cu II) complex of ketoprofen-salicylhydrazone (FPA-306) (Hurtado et al., 2018; Gou et al., 2021). Cuproptosis has only recently been identified, which was deem to be a new unique form of cell death (Polishchuk et al., 2019; Aubert et al., 2020; Dong et al., 2021; Ren et al., 2021; Tsvetkov et al., 2022). The method of cell death leads to the gathering of acylated protein and downregulating the iron-sulfur protein *via* the binding of copper to lipid acylated components of the TCA. The procession results in proteotoxicity and cell death in the end (Koh et al., 2017). Interestingly, there were several clinical trials that have been performed with the copper ionophore micromolecular anti-tumor drugs Elesclomol (Soma et al., 2018). The results failed to get a satisfactory outcome. LncRNAs have been widely recognized to have a deep connection with tumor progression, including BC (Cao et al., 2019; Luo et al., 2019). However, rarely studies have found the regulatory role in BC.

In our present study, 69 CRLs were identified from co-expression of lncRNAs and CRGs. With the analysis of univariate and multivariate Cox regression, 3 CRLs with prognostic value, including AL590428.1, AL138756.1 and GUSBP11, were achieved and a prognostic model was developed. The results from ROC, OS, PFS, nomogram and heatmap indicated the signature of 3 CRLs distinguished prognostic features with good accuracy in two subgroups in BC patients. Meanwhile, clinical outcomes in BC patients were predicted as independent prognostic factors. Meaningfully, AL590428.1 was investigated in cancer firstly. It is reported that AL138756.1 participated in predicting the prognosis of colon adenocarcinoma as a prognostic indicator (Zhou et al., 2020). GUSBP11 has been found to regulate the progression of tumor, including triple negative breast cancer and nasopharyngeal carcinoma (Wu et al., 2022; Zhang et al., 2022). Recent research has found that GUSBP11 was contained in a machine learning-based computational network for an indicator of immune infiltration of tumor microenvironment (Zhou et al., 2021). Herein, we further elucidated the correlation between GUSBP11 and BC. Then, signaling receptor activator activity, external side of plasma membrane, and positive regulation of cell activation were found in the GO enrichment. The KEGG pathway analysis revealed cytokine-cytokine receptor interaction, viral protein interaction with cytokine and cytokine receptor and PI3K-Akt signaling pathway. Therefore, the results suggested that disorder interactions between bioactive molecules and cellular signaling pathway severely promote the progression and generate poor clinical prognostic outcomes.

Furthermore, the correlation between TMB, immune function and risk scores was analyzed in BC. TMB was commonly regarded as an indicator for immune checkpoint blockade (ICB) in BC, lung cancer and melanoma (Fusco et al., 2021; Jardim et al., 2021; McGrail et al., 2021). Although the lack of significant differences between two subgroups (*p* > 0.05), the frequency of mutation genes in subgroups has changed a lot and survival time was significantly prolonged in low TMB (*p* < 0.05). We found the mutation of TP53 and TTN was increased but KMT2D, MUC16 and ARID1A was decreased in high-risk group. An increasing research has demonstrated TP53 and TTN have an important effect on the promotion of tumor (Mogi and Kuwano, 2011; Fu et al., 2019; Shen et al., 2020; Siracusano et al., 2020). According to our results, the same conclusion keeps in line with previous researches. Moreover, KMT2D and ARID1A were found to be decreased in expression (Garczyk et al., 2018; Sun et al., 2019b), and MUC16 can be regarded as potential surrogate biomarker of poor prognosis and unique molecular signature (Cotton et al., 2017). Here, low-risk group showed higher mutation levels of KMT2D and ARID1A than high-risk group. In line with previous studies, our results are also valid. We evaluated the prognosis of BC patients in immune-related functions and contribute to figure out the relationship between lncRNAs and immune functions. The analysis of immune-related functions implied all members of the risk signature were closely related to the antigen-presenting of tumor. Meanwhile, the results of the TIDE algorithm indicated a high-risk group received more benefit from immunotherapy, which keeps in line with previous conclusions. Due to the advent of Bacille Calmette Guerin (BCG), BC is one of the earliest cancers where the concept of immunotherapy was proposed (Vasekar et al., 2016). While it is true, recent advances have revealed multiple molecular mechanism as a prevalent tumor therapy, such as CD24/Siglec-10, EMT, PD-L1/PD-1, C/EBPβ transcription factors and hypoxia/HIF-1α (Marigo et al., 2010; Vaupel and Multhoff, 2018; Jiang et al., 2019; Jiang and Zhan, 2020; Yin and Gao, 2020). Although LncRNAs itself do not code proteins, immunotherapy and immune responses have been identified to participate (Jiang et al., 2021). The interactions between lncRNAs and cancer immunotherapy involved various immune cells in tumor microenvironment (Luo et al., 2020), which is consistent with our analyses of immune-related function. Besides, the pRRophetic algorithm was conducted to screen for potential anti-tumor drugs *via* the analysis of sensitivity and correlation of those drugs. Part of those have been applied to other cancers. Despite the drugs mechanisms of impact on BC need to be figure out, it provides a new insight on drawing up a therapeutic schedule.

In brief, a CRLs signature of BC was constructed. In the light of different calculated risk scores, two subgroups were divided and the relationship was analyzed between TMB and subgroups, immunotherapy and drug sensitivity. Therefore, a novel strategy for predicting survival status and optimizing clinical therapy for BC has been developed in our study.

## Data Availability

The datasets presented in this study can be found in online repositories. The names of the repository/repositories and accession number(s) can be found in the article/Supplementary Material.

## Abbreviations

BC, bladder cancer; CRLs, cuproptosis-related lncRNAs; CRGs, Cuproptosis-related genes; TMB, tumor mutation burden.
