# Supplementary figures and images for: A novel cuproptosis-related lncRNA signature predicts prognosis and therapeutic response in bladder cancer
Source: Front Genet. 2023 Jan 4;13:1082691. doi: 10.3389/fgene.2022.1082691 (PMC9845412; doi:10.3389/fgene.2022.1082691)

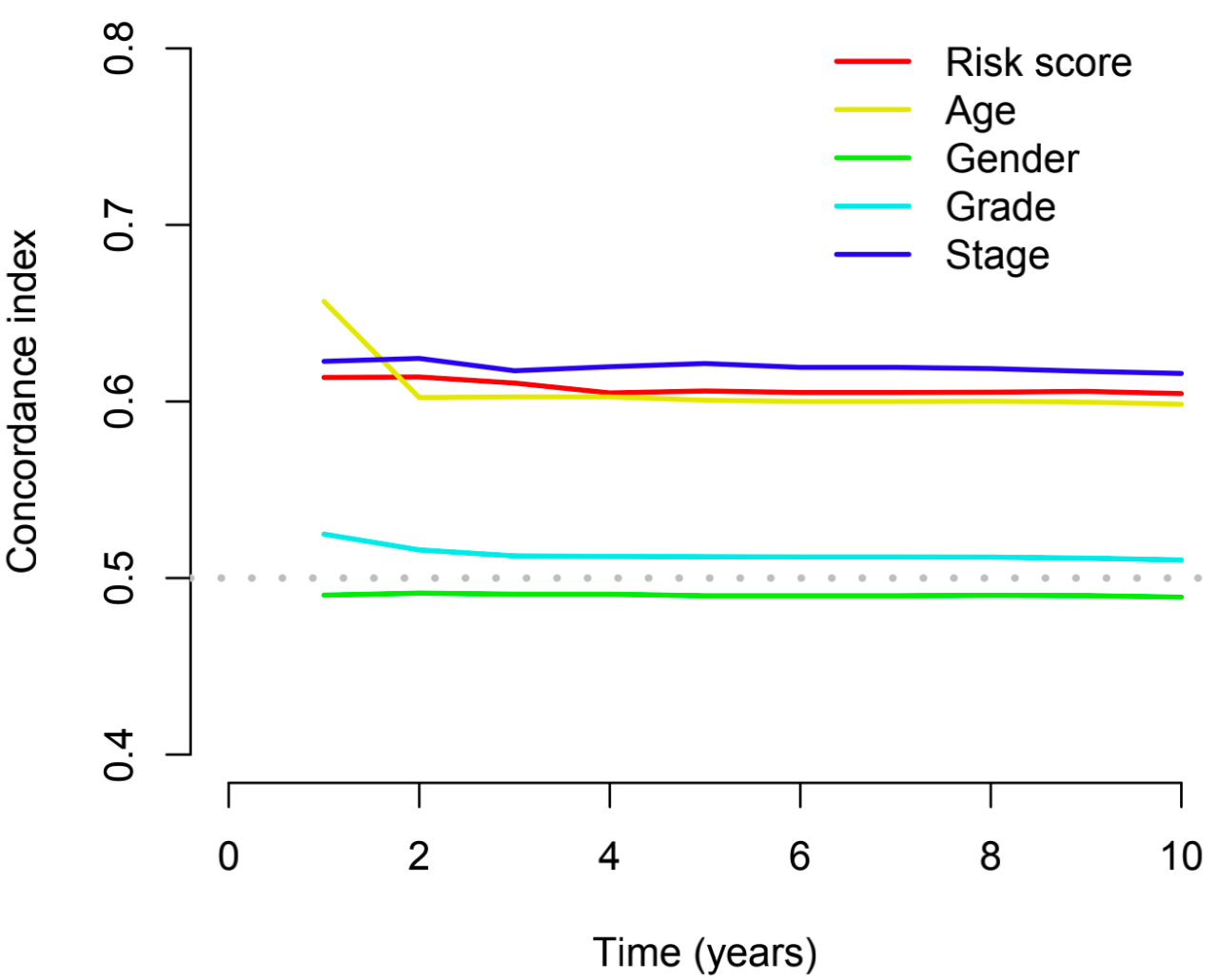

Supplement: Supplementary file 3 [file Image2.PNG]

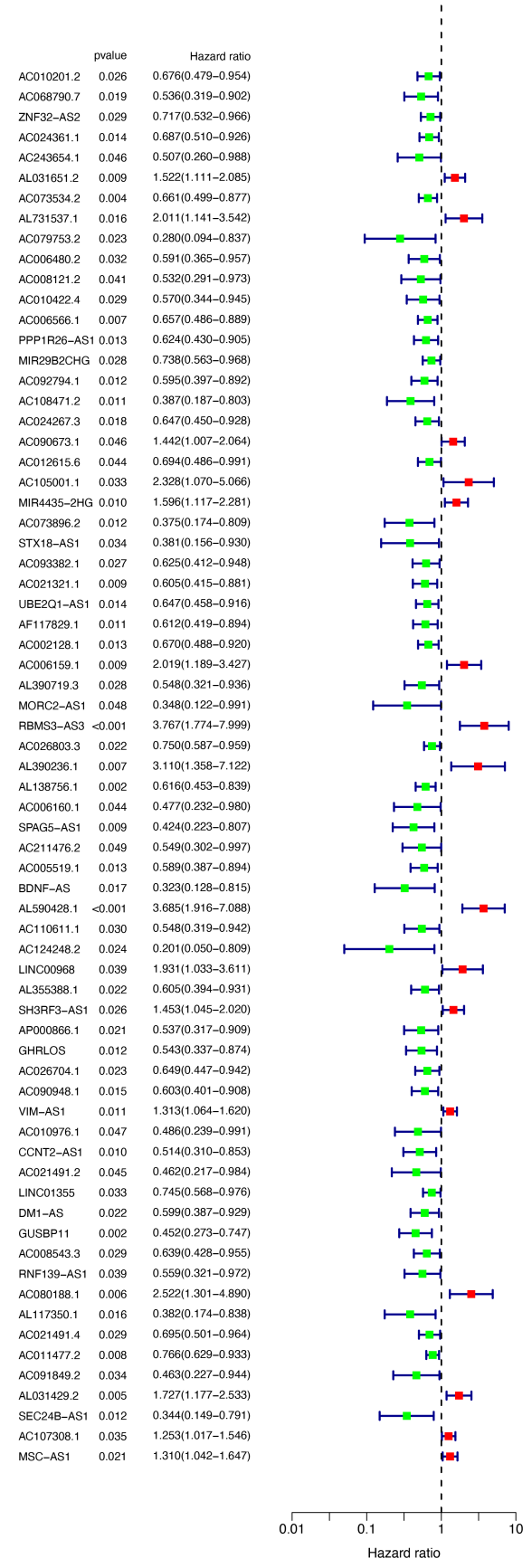

Supplement: Supplementary file 5 [file Image1.PNG]
